# Psychogeriatric research during COVID-19 pandemic: qualitative analysis of participant views

**DOI:** 10.1017/S1041610220001179

**Published:** 2020-06-11

**Authors:** Hillary D. Lum, Kalpana P. Padala, Kim T. Dean, Prasad R. Padala

**Affiliations:** 1 Geriatrics Research Education and Clinical Center, VA Eastern Colorado Healthcare System, Aurora, CO, USA; 2 Division of Geriatric Medicine, University of Colorado School of Medicine, Aurora, CO, USA; 3 Geriatric Research Education, and Clinical Center (GRECC), Central Arkansas Veterans Healthcare System (CAVHS), Little Rock, AR, USA; 4 Department of Geriatrics, University of Arkansas for Medical Sciences (UAMS), Little Rock, AR, USA; 5 Department of Psychiatry, University of Arkansas for Medical Sciences (UAMS), Little Rock, AR, USA

Conducting clinical research during pandemics is critical not only for the management of the ongoing pandemic but also for future pandemic preparedness (Gobat *et al*., 2018). The Department of Veterans Affairs (VA), a large integrated health organization and a major sponsor of patient-centered research, is a key player in shaping the conduct of psychogeriatric research in the USA. Sponsored by the VA Office of Research and Development (ORD), VA investigators are involved in over 7,000 funded research projects and often focus on psychogeriatric patient-centered research due to the demographics of Veterans and their family caregivers (ORD, 2020). Hence, it is important to understand how the ORD is handling clinical research during the current pandemic.

The current pandemic is nothing like what the existing research infrastructure has previously witnessed. On March 11, 2020, the World Health Organization characterized coronavirus disease 2019 (COVID-19) as a pandemic. At that time, there were approximately 118,000 cases in 114 countries, and 4,291 people had died worldwide due to COVID-19. There were 1,215 total COVID-19 cases in the US; recommended coronavirus mitigation efforts included self-isolation and avoidance of healthcare centers where symptomatic patients congregate for medical care and where patient-centered research is typically conducted (CDC, 2020). Since the pandemic declaration of COVID-19, there has been rapid spread of COVID-19 in the world and clear recognition of the burden of illness and excess mortality among older adults, especially those with cognitive impairment, frailty, and residing in nursing homes (Shahid *et al*., 2020). As we experience ongoing rapid changes in clinical care related to the pandemic, views regarding conducting clinical research involving older adults are also evolving (Nicol *et al*., 2020).

Despite the benefits of patient-centered research, a pandemic brings huge disruptions in safely conducting clinical research (Gobat *et al*., 2018). Therefore, VA ORD prioritized the health and safety of study participants and appropriately deemed clinical research visits as nonessential during the COVID-19 pandemic, in line with the US Centers of Disease Control and the US National Institute of Health (CDC, 2020, NIH, 2020). However, at the same time there are calls for using the existing VA clinical research infrastructure for COVID-19-related research. While researchers have discussed challenges to conducting clinical trials during COVID-19, including efficient accrual and randomization, intervention adherence and delivery, and outcome collection (McDermott and Newman, 2020), less is known about the perspectives of older participants and their caregivers related to their involvement in patient-centered research during a pandemic. This commentary highlights implications for psychogeriatric research based on the perspectives of older adults and caregivers in the context of the early days of the COVID-19 pandemic.

In March of 2020, recognizing the importance of rapidly engaging patients and caregivers during the COVID-19 pandemic, we briefly surveyed 51 participants who were enrolled in several ongoing psychogeriatric research studies pertaining to mild cognitive impairment, as well as major neurocognitive disorder with and without behavioral problems at the VISN 16 Little Rock Geriatric Research Education and Clinical Center, Little Rock, Arkansas, US (Padala *et al*., 2020). This was before the VA ORD released guidance to temporarily halt all new enrollments and in-person research visits unless such a halt would be detrimental to the participants. We spoke with participants either during their in-person visit or via phone if they had canceled/postponed the visit. We asked open-ended questions to explore participants’ potential concerns about handling the pandemic, the VA healthcare system’s COVID-19 screening process, and general advice to improve the process of healthcare visits (e.g. what helped you decided to attend this appointment or skip it? Do you have any concerns about how the VA is handling the situation? What advice do you have to improve our processes? Do you prefer an in-person visit or a telephone visit?) We also asked about their primary source of COVID-19-related information and the reasons they preferred that source over others. A total of 51 participants were approached all of whom responded. Of these, 31 were research participants and 20 were caregivers with a mean age of 69.3 (±9.4) years and a mean education of 12.8 (±2.3) years, 53% were males and 65% were Caucasian. While some responses from these older adult psychogeriatric research participants were expected, other input was surprising.

## Deciding whether to attend visits: influenced by trust in the healthcare system

Regarding the decision to come to the VA healthcare facility for a scheduled research appointment, key positive factors were trust in the VA system, extra screening at the facility entrance related to COVID-19, and the fact that the research visit was not in a group format. For example, participants noted, “I am in a safe spot with the VA,” “We have limited going around. We did stock up. We feel safe coming to the VA,” and “Extra screening was great.” The low number of cases in the community also seems to have played a role in the decision process, “Low cases in Arkansas, no need to panic.” Related to trusting the VA, participants felt that the VA was prepared, professional, and acting in the Veterans’ best interests.

Some older Veterans voiced concerns about participating in research during the COVID-19 pandemic that were expected, while other concerns suggested the participants were influenced by myths or misconceptions. Multiple expected responses focused on risks for increased infection, underlying conditions or frailty of the participant making him/her more vulnerable for infections and worry about exposing older parents. A concerning reason for deciding not to participate in the research visit was “I am concerned that researchers were trying to give me coronavirus as a guinea pig.” Some lack of concern related to the pandemic seemed to be based on myths or mis-information, “I have terrific immune system as I work in the schools,” and “It does not affect black people.”

## Older adults’ COVID-19 information sources

Close family members and friends and traditional news media were the common resources that participants accessed for COVID-19 information. Three respondents each obtained information from the VA social media/newsletter and the US Centers for Disease Control newsletter. Several participants expressed concern that social media may be contributing to spread of unauthenticated information and that public should turn to experts.

## In-person versus telephone research visits

When given a choice of conducting the research visit over the telephone, half of the participants preferred a telephone visit over in-person visit. Several participants had variable levels of acceptance related to the choice of telephone visits for research purposes. One participant advised, “Don’t go out. Glad that you are offering televisit,” reflecting the decision some made to limit infection risk was by deferring in-person visits. However, many others were not comfortable with telephone visits due to hearing impairment, uncertainty about the quality of care, and concern about when they would get their next appointment if they canceled the appointment. One participant felt that he would be more wary of research if it was all done over the phone.

### Implications

The voices of older research participants and their caregivers in the design and conduct of patient-centered research are of utmost importance. Similarly, the perspectives of these research participants are also important in public health emergencies, like pandemics. We hope to elevate the perspectives of research participants and caregivers enrolled in psychogeriatric studies during the early days of the COVID-19 pandemic. These individuals offer real-world perspectives on decision-making related to research participation that has implications for research during highly disrupted times. While many Veterans expressed high levels of trust in the VA healthcare system, they also had tangible concerns related to risk of infection and logistics of how to continue participation in research during the pandemic. Education about COVID-19 infection, transmission, and universal precautions should be provided continually to both patients and caregivers (Wang *et al*., 2020).

An important implication is that communication from research staff is key when enacting research-specific protocol changes (e.g. telephone visits instead of in-person visits) or new facility-level processes (e.g. new health screenings). Clear communication about current status and any changes from the research staff may help prevent dropout and/or nonadherence by reassuring the participants that their involvement in the study remains important, even during the pandemic (McDermott and Newman, 2020). One example noted in the study was the need for clear communication about extra screening at in-person visits. For example, a phone call to describe a new health screening process might prepare participants and could reinforce accurate information about the pandemic. Improving the screening processes based on concerns such as not reusing fomites such as pens to sign-in names needs to be undertaken. One simple solution would be for the healthcare staff member to document the temperature and the needed details of those being screened.

Given the importance of outcome collection, researchers may plan to transition to collecting outcomes remotely such as by telephone or online (McDermott and Newman, 2020). The Department of VA has invested significantly in telehealth technology, but the research enterprise may lag behind the clinical division due to regulatory burdens (Young *et al*., 2011). This study, however, recognizes that some older Veterans and caregivers may have barriers to these modalities and emphasizes the importance of communicating the rationale and exercising as much flexibility as is safely possible based on participants’ needs. Using VA social media to make user-friendly promotional videos about telehealth use might improve its reach. It also appears that some participants were concerned about getting the next appointment if they were to cancel the current one. Reassuring participants about clinic availability (for research or clinical purposes) and process would be helpful.

Family members and caregivers were noted to be the primary source of information for most respondents. It is imperative to work on messaging the caregivers about the pandemic. Education is needed because studies have shown that African Americans are at higher risk of COVID-19-related mortality than other ethnicities (Yancy, 2020). We heard that some respondents were concerned that social media contributes to spread of unauthenticated information. Thus, providing a clearing house of credible information sources would be helpful. For example, many VA medical centers have started telephone-based hotlines to mitigate false information about COVID-19 called, “Myth Busters.” Similar hotlines for research participants would be helpful.

In conclusion, we seek to elevate the opportunity for psychogeriatric researchers to understand the needs of older adult research participants and adapt research studies based on input from patients and caregivers. Even as participants may place a high level of trust in their healthcare system and its preparedness in combating the COVID-19 pandemic, clear communication from the research staff may help prevent dropout or nonadherence. Researchers need to reassure participants that their involvement in the study remains important, even during the pandemic. Additionally, because family members and caregivers often serve as a primary source of information, it is imperative to work on broad and accurate information and messaging about the pandemic.
